# Takotsubo Cardiomyopathy: A New Perspective in Asthma

**DOI:** 10.1155/2015/640795

**Published:** 2015-07-12

**Authors:** Fady Y. Marmoush, Mohamad F. Barbour, Thomas E. Noonan, Mazen O. Al-Qadi

**Affiliations:** ^1^Memorial Hospital of Rhode Island, Alpert Medical School, Brown University, Pawtucket, RI 02860, USA; ^2^Harvard Medical School, USA

## Abstract

Takotsubo cardiomyopathy (TCM) is an entity of reversible cardiomyopathy known for its association with physical or emotional stress and may mimic myocardial infarction. We report an exceedingly rare case of albuterol-induced TCM with moderate asthma exacerbation. An interesting association that may help in understanding the etiology of TCM in the asthmatic population. Although the prognosis of TCM is excellent, it is crucial to recognize beta agonists as a potential stressor.

## 1. Introduction

Takotsubo cardiomyopathy (TCM) has been recognized more recently as an entity of reversible cardiomyopathy. It is also known as stress-induced cardiomyopathy for its strong association with physical or emotional stress. The clinical presentations and electrocardiographic changes are nonspecific. Furthermore, cardiac biomarkers are frequently elevated which makes the differentiation between TCM and acute myocardial infarction challenging. We are reporting a case of albuterol-induced TCM associated with moderate asthma exacerbation due to excess albuterol use. This case report along with few other reports in the literature sheds light on an unrecognized etiology for TCM in patients with asthma exacerbation.

## 2. Case Description

**Figure 1:**
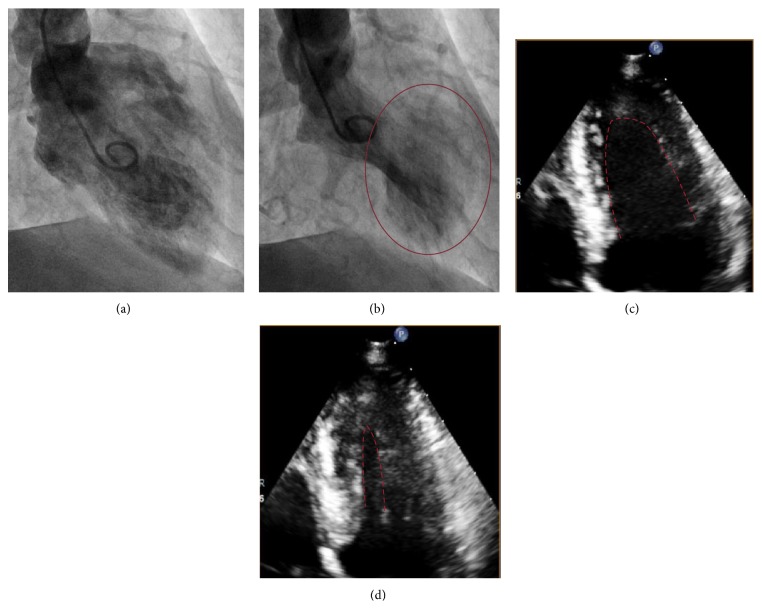
Left ventricular catheterization shows (a) the end of diastole and (b) apical ballooning at the end of systole. Transthoracic echocardiogram shows (c) LV end of diastole and (d) LV end of systole (no apical ballooning).

An 80-year-old woman with past medical history of asthma, hypertension, and hyperlipidemia presented with progressively increasing shortness of breath and wheezing associated with left-sided, constant, nonradiating chest pain that was not exertional and started few hours prior to her presentation. She used her rescue albuterol inhaler several times. She denied any fever, chills, or cough. She has never smoked in the past. Her only medication for asthma is albuterol inhalation as needed. Her chest pain resolved spontaneously several minutes before arrival to the emergency room. On examination, she had nontoxic appearance and was in mild distress. She was afebrile and the blood pressure was 145/64 mmHg and she had a pulse of regular 100 beats per minute and respiratory rate of 22 breaths per minute. Oxygen saturation was 93% on ambient air. Chest exam was remarkable for prolonged expiratory phase and scattered expiratory wheezes but no rhonchi or accessory muscle use. Laboratory findings on admission showed white blood cell count 6,200/mm^3^, hemoglobin 13 g/dL, and troponin I mildly elevated at 0.05 ng/mL (normal reference < 0.030 ng/mL). B natriuretic peptide was 55 pg/mL. Chest radiograph was normal. Electrocardiogram (EKG) was remarkable for left atrial enlargement and new left bundle branch block (LBBB). Initial management for asthma exacerbation included intravenous methyl-prednisone and albuterol/ipratropium along with continuation of aspirin. Though the shortness of breath improved after a few hours, the patient experienced recurrent chest pain relieved with nitroglycerine. The second troponin rose to 0.422 ng/mL and peaked at 1.112 ng/mL on serial measurements with no changes on EKG. Management for acute coronary syndrome was initiated including aspirin, clopidogrel, statins, and intravenous heparin. Echocardiography demonstrated ejection fraction of 60–65% with hypokinesis of the left ventricular apex and distal septum. Cardiac catheterization was performed revealing normal epicardial coronary arteries with evidence of apical ballooning (Figures 1(a) and 1(b)). Based on these findings, she was diagnosed with TCM. Eventually, the troponin trended down with no recurrence of chest pain or dyspnea during hospitalization. Three months later, the patient had persistence of left bundle branch block and repeat echocardiography (Figures 1(c) and 1(d)) showed complete resolution of apical ballooning with preserved ejection fraction.

## 3. Discussion

Stress-induced cardiomyopathy also known as Takotsubo cardiomyopathy (TCM) was first reported in Japan in 1990 by Sato et al. Tako-tsubo is a Japanese term that describes the octopus trap, referring to the apical ballooning morphology of the left ventricular apex associated with the condition. TCM is an acute but reversible subtype of cardiomyopathy that may mimic acute myocardial infarction with no evidence of obstructive epicardial coronary artery disease. It is usually preceded by acute physical or emotional stress such as grief, exacerbation of chronic illnesses (e.g., asthma), or use of exogenous catecholamines. TCM has a distinctive morphology of hypokinesis of the left ventricular mid- and apical segments with hyperkinesis of the basal segments leading to distal ballooning of the apex [1]. In a single-center study, approximately 2.2% of patients presenting with ST segment elevation had TCM [2].

We report an exceedingly rare case of albuterol-induced TCM. To our knowledge, there were only 5 similar cases reported in literature [3–7]. Although our patient presented with asthma exacerbation which may count as an acute stressor, there was no evidence of severe stress as in status asthmaticus or respiratory failure as previously reported in two cases [5, 6]. The likely mechanism of TCM in our case is mediated through the *β* agonist action of albuterol. Although *β*2 agonists are highly selective, they may lose their selectivity at high doses [8]. Furthermore, Paur et al. showed that there is a myocardial depressive effect through catecholamines-specific *β*2 adrenergic receptor inhibitory signaling in a rat model with regional preference to the apex given the ventricular apical-basal *β*2 adrenergic receptor gradient [9]. Moreover, TCM has been described after the use of other *β* agonists [10].

Another postulated mechanism for TCM is microvascular spasm with subsequent myocardial apical stunning [11, 12], which explains the extent of myocardial dysfunction beyond a single coronary territory and may explain the presence of left bundle branch block. Although coronary microvascular spasm is less likely to explain TCM in asthmatics [13–15], our patient had persistence of left bundle branch block in the absence of reduced ejection fraction which may indicate an underlying microvascular ischemia contributing to the presentation.

Spasm of multiple epicardial vessels or aborted infarction of a long wrap around left anterior descending artery is not supported by angiographic and intravascular ultrasonographic studies.

Our patient fits into the demographic profile of TCM described in literature [1, 2, 12, 16–20]. Most patients were postmenopausal women (82% to 100% of patients). Chest pain was observed in 50–70% of cases while the majority had typically mild elevations in troponin levels as our patient had. Our case shows evidence of new left bundle branch block which was described to occur in 9% of patients with TCM on presentation, in particular, our patient's age group [21]. Given the nonspecific clinical features, the diagnosis of TCM is usually made after excluding acute coronary events. In addition, the modified Mayo Clinic criteria for diagnosis of TCM are frequently used. Patients must meet all four criteria: (a) transient left ventricular midsegments hypokinesis or akinesis (with or without apical involvement) that extends beyond a single vessel distribution, with or without a stressor; (b) absence of occlusive coronary disease; (c) evidence of myocardial injury (new EKG abnormalities or elevation in troponin); and (d) exclusion of pheochromocytoma or myocarditis. Treatment of TCM is supportive and includes the standard therapy for cardiomyopathy (angiotensin converting enzyme inhibitor and beta blockers). Diuretics are used to manage pulmonary edema and other manifestations of volume overload. Mechanical circulatory support devices (e.g., intra-aortic balloon pump) can be used in cases complicated by cardiogenic shock. When left ventricular thrombus is present, anticoagulation is indicated.

The prognosis of TCM is excellent with most patients recovering completely within 4–8 weeks. Recurrence may occur in 1.5% of patients and can be reduced by angiotensin converting enzyme inhibitors [22]. The effect of beta blockers on recurrence is less defined. Fatality from TCM is rare and occurs in less than 3% of patients [23].

## 4. Conclusion

Although the prognosis of TCM is excellent, it is crucial to recognize beta agonists as a potential stressor, especially in patients with acute asthma attacks. Despite increasing awareness of beta agonist-induced TCM, the diagnosis of TCM remains that of exclusion.
